# Does training level affect the accuracy of visual assessment of capillary refill time?

**DOI:** 10.1186/s13054-019-2444-3

**Published:** 2019-05-06

**Authors:** Koichiro Shinozaki, Lee S. Jacobson, Kota Saeki, Naoki Kobayashi, Steve Weisner, Julianne M. Falotico, Timmy Li, Junhwan Kim, Joshua W. Lampe, Lance B. Becker

**Affiliations:** 1 The Feinstein Institute for Medical Research, Northwell Health System, 350 Community Dr., Manhasset, NY 11030 USA; 2 Department of Emergency Medicine, North Shore University Hospital, Northwell Health System, Manhasset, NY USA; 3 Nihon Kohden Innovation Center, Cambridge, MA USA; 4 Nihon Kohden Corporation, Tokyo, Japan; 5 ZOLL Medical, Chelmsford, MA USA

Capillary refill time (CRT) measured at the bedside is widely promulgated in critical care and intensive care medicine [1, 2]. However, traditional CRT measurements are relatively subjective [3], and the accuracy is questionable given that clinicians use the naked eye to perform these visual assessments [4, 5]. The purpose of our study was to evaluate the accuracy of visually assessed CRT among observers who have different training levels.

**Fig. 1:**
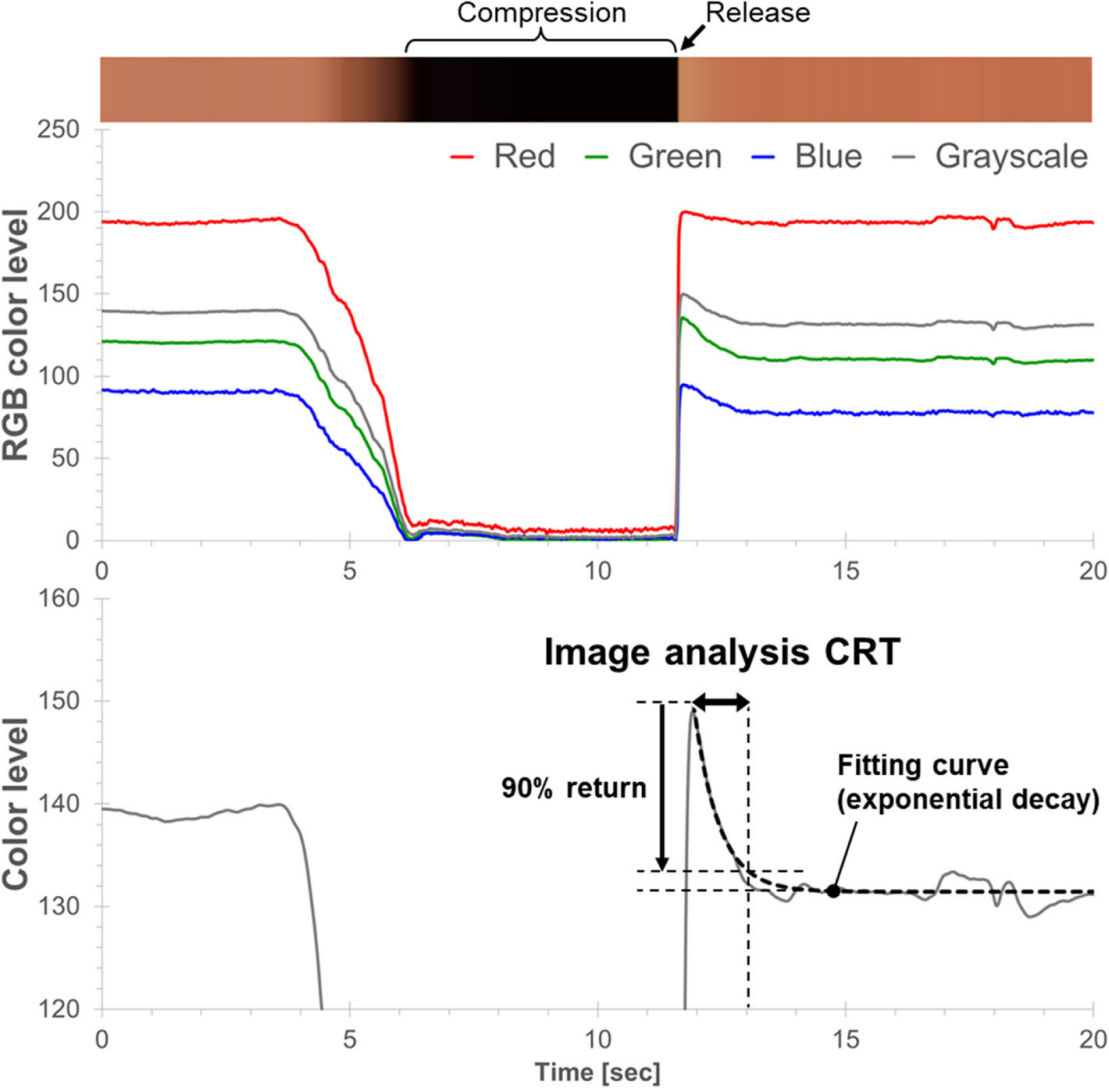
Image analysis CRT. The recorded videos were used thereafter in a separate setting to calculate CRT via image software analysis (image analysis CRT). Averaged color of the fingernail area was extracted from the digital video file and the color change was represented as RGB waveforms. And then, the RGB waveforms were converted to a grayscale waveform. A curve fitting the returning phase of the grayscale waveform was modeled as an exponential decay using the least squares method. The time to achieve 90% return of fitting curve was reported as “image analysis CRT”

Fingernail compression and release videos were recorded from patients in the emergency department (ED) at a suburban, quaternary care teaching hospital in New York. We used our image analysis software to analyze the corresponding fingernail video to calculate patient’s CRT (Fig. 1). Nine clinicians and two non-clinicians voluntarily participated as observers to review the videos. Videos from 20 patients were displayed on a screen three times in random order, for a total of 60 videos. The observers watched each fingernail video and pressed a time switch when they deemed the fingernail color had returned to its baseline state. The truth of visually assessed CRT was evaluated by using a correlation of the numbers between the image analysis and the visual assessment. We also sought to determine the intra-observer reliability to evaluate the precision of visual assessments.

**Fig. 2:**
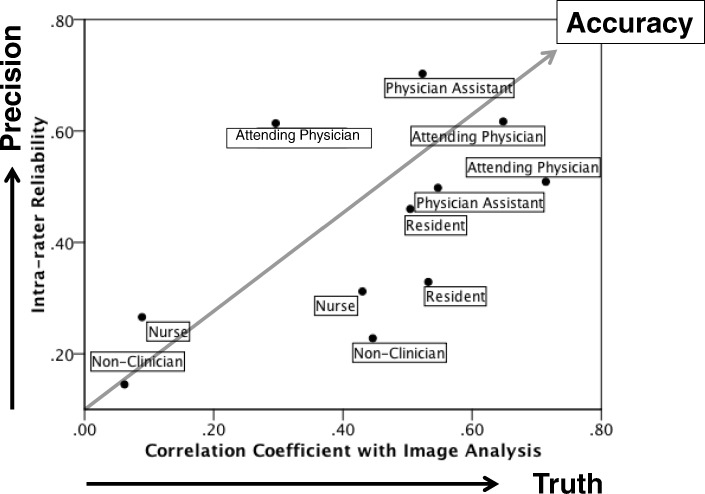
Scatter plot showing intra-rater (observer) reliability of video assessment CRT as a function of correlation coefficient of video assessment CRT with image analysis CRT. Attending physicians (22 and 28 years of ED work experience), residents (3 years of ED work experience), nurses (2 years of ED work experience), and physician assistants (1 and 2 years of ED work experience) participated in the study. Six clinicians were actively performing CRT assessments in their clinical work. Observers, who showed higher correlation with image analysis CRT, demonstrated higher intra-rater reliability, and there was a strong correlation between these coefficient values (*r* = 0.72, *p* < 0.05)

Image analysis of CRT of 20 ED patients ranged from 0.47 to 7.98 s, with a mean of 2.44 ± 2.09 s. The highest intra-observer reliability among the three visual assessment times was displayed by one of the physician assistants (0.70 for single measure and 0.88 for average measures); however, it was also as low as 0.15 for a single measure and 0.34 for average measures by one of the non-clinicians. Intra-observer reliability was the highest in attending physicians and physician assistants, followed by residents, nurses, and non-clinicians. The mean intra-observer reliability of the clinicians was higher than the non-clinicians (0.46 vs. 0.25, *p* < 0.05). Figure 2 shows intra-observer reliability of the video assessment as a function of correlation coefficient of video CRT assessment with image CRT analysis. Observers, who showed a higher correlation with image CRT analysis, demonstrated higher intra-observer reliability, and there was a strong correlation between these coefficient values (*r* = 0.72, *p* < 0.05).

Visual assessment of CRT is variable. Personal work experience may help improve both truth and precision of CRT assessments and increase the accuracy among individual observers. Therefore, training level appears to be an important factor that affects the reliability of visual CRT assessment.
